# Establishment of a rapid method for the detection of *Brucella* canis based on recombinase-mediated thermostable nucleic acid amplification technology

**DOI:** 10.3389/fcimb.2024.1493492

**Published:** 2025-01-03

**Authors:** Shao-Zheng Song, Zi-Yuan Li, Yuan-Yuan Liu, Ying-Chao Wu, Kang-Ying Yu, Zhengyi He

**Affiliations:** ^1^ School of Health and Nursing/Department of Basic, Wuxi Taihu University, Wuxi, Jiangsu, China; ^2^ School of Health and Nursing/Department of Nursing, Wuxi Taihu University, Wuxi, Jiangsu, China; ^3^ Department of Internal Medicine, Jiangyin Lingfeng Pet Hospital, Wuxi, Jiangsu, China; ^4^ The First Affiliated Hospital of Gannan Medical University, Ganzhou, Jiangxi, China

**Keywords:** recombinase, thermostatic, nucleic acid amplification, rapid detection, Brucella canis

## Abstract

**Objective:**

To establish a rapid detection method for canine *brucellosis* using recombinase-aided amplification (RAA) technology.

**Methods:**

The outer membrane protein 25 gene fragment (Omp25) of *Brucella* canis was targeted. Primers and fluorescent probes were designed and synthesized, and recombinant plasmids were constructed as standards. The RAA assay was optimized by screening primers and establishing a fluorescent reaction system. Sensitivity was analyzed using plasmid standards with varying copy numbers. Specificity was tested using genomes from *Brucella* canis, *Brucella* suis, *Brucella* melitensis, *Brucella* abortus, *Staphylococcus aureus, pathogenic Escherichia coli, Salmonella enteritidis, Shigella* spp.*, Proteus mirabilis, and Listeria monocytogenes.* Reproducibility was evaluated using plasmid standards from the same and different batches.

**Results:**

The optimized RAA system used primers bOmp25-F2/bOmp25-R2 and probe bOmp25-P, with a constant reaction temperature of 39°C for 15 minutes. The detection sensitivity was 1 copy/μL. No cross-reaction was observed with other *Brucella* species or pathogenic bacteria, indicating high specificity. Intra-batch variability was below 1.00%, and inter-batch variability was below 2.00%. The positive detection coincidence rate of RAA was significantly higher than that of commercial real-time fluorescence quantitative PCR (100% VS 86.96%, P<0.05).

**Conclusion:**

The RAA-based rapid detection method for *Brucella* canis is suitable for clinical rapid testing. It offers advantages such as quick detection, high sensitivity, strong specificity, and good reproducibility. This method provides new insights for the rapid detection of canine *brucellosis* and the precise diagnosis of other pet diseases, making it suitable for promotion and application.

## 1 Introduction

Canine *brucellosis*, also known as Mediterranean flaccid fever, Malta fever, or wave fever, is a zoonotic systemic infection caused by *Brucella* abortus. It is characterized by prolonged fever, excessive sweating, joint pain (arthralgia), hepatosplenomegaly, and may also lead to reproductive disorders (Djokic et al., 2023; Weese et al., 2020; Ishihara et al., 2024).Since 1966, when *Brucella* canis was first detected in the tissues and vaginal secretions of diseased dogs by Carmichael et al., the degree of harm and prevention and control of the disease have been receiving attention (Sebzda and Kauffman, 2023), which is classified as a legally-reported animal disease by the World Health Organization (WOAH), and controlled as a Class B infectious disease by the Law of the People’s Republic of China on Prevention and Control of Infectious Diseases in our country. *Brucella* canis is a kind of Gram-positive bacteria parasitized in the host cells, highly infectious, with various transmission routes and wide prevalence. There are nearly 200 countries and regions where this disease exists, which is a great danger to public health, not only jeopardizing human life and health, but also seriously threatening the development of canine breeding production, and causing great economic losses to the majority of pet enthusiasts (van Dijk et al., 2021; Donoghue et al., 2024; Santos et al., 2021). In recent years, with the continuous expansion of the scale of dog breeding in China, the number of dogs has increased substantially, and the epidemiological complexity and variability have led to a year-on-year increase in the incidence of canine *brucellosis*, so there is a need to increase the precise monitoring and comprehensive prevention and control of the disease (Tymczak et al., 2022; Moore et al., 2024). Studies have shown that early and accurate differential diagnosis significantly enhances the clinical treatment, prevention, and control of canine *brucellosis* (Schiavo et al., 2024). To better prevent and control canine *brucellosis*, it is essential to actively develop and establish a detection method that is accurate, specific, and highly sensitive.

Once a dog is infected with *Brucella*, the infection is typically latent and lacks clear clinical symptoms, making diagnosis challenging based on symptoms alone. Therefore, laboratory-specific diagnostic methods are required to confirm the infection (Camargo-Castañeda et al., 2021). At present, the detection methods of *Brucella* are mainly pathogenic diagnosis, serological diagnosis and molecular biology diagnosis, but these methods have the defects of requiring special laboratories, high safety risk, complicated operation, long time-consuming detection, low sensitivity and high cost of instrumentation and equipment (Süer et al., 2023; Mol et al., 2020). The national diagnostic standard for detecting *Brucella* in China, Bruce Ladder PCR (GB/T 18646-2018), utilizes polymerase chain reaction (PCR) to identify strains at the genetic level and reduce diagnostic risk. However, this method requires isolation and cultivation of the pathogen, has limited sensitivity, and demands high technical expertise, making it unsuitable for widespread use in rapid field testing at the grassroots level (Ye et al., 2022). In recent years, with the rapid development of molecular biology detection technology, based on the application of Recombinase aided isothermal nucleic acid amplification (RAA) detection technology. This technology is a new type of nucleic acid detection technology, with the help of recombinase, polymerase and single-stranded binding protein, etc., which can be used as a substitute for PCR technology, and can realize rapid amplification of target nucleic acid fragments within 20 min under constant temperature, and combined with fluorescent probes, it can be detected instantly (Liu et al., 2024), which has the advantages of convenient detection, rapidity, sensitivity and precision, and has begun to be applied to the detection of various pathogens in various animal epidemics step by step (Qin et al., 2019; Sutipatanasomboon et al., 2024; Rong et al., 2023; Sun et al., 2024),which is applied in the rapid detection and diagnosis of canine *brucellosis*, and has an important role in the comprehensive prevention and control of the disease.

In addition, the outer membrane protein 25 (Omp25) gene sequence of *Brucella* Canis is highly conserved and can be used to identify the target sequences of different strains. Therefore, in order to rapidly and accurately detect canine *brucellosis*, the Omp25 gene fragment was selected as the target sequence in this study. In addition, the outer membrane protein 25 (Omp25) gene sequence of *Brucellosis* Canis is highly conserved and can be used as the target sequence for the identification of different strains. Therefore, in order to rapidly and accurately detect canine *brucellosis*, the Omp25 gene fragment was selected as the target sequence in this study. To enable rapid and accurate detection of canine *brucellosis*, this study aims to target the *Brucella* canis outer membrane protein 25 gene fragment (Omp25) for the design of primers and fluorescent probes, optimizing reaction conditions to establish a rapid recombinase-aided amplification (RAA) detection method. This approach not only enhances the field-based detection of canine *brucellosis* but also provides a novel tool for the on-site diagnosis of other animal diseases and supports clinical veterinarians in the precise diagnosis of pet diseases.

## 2 Materials and methods

### 2.1 Materials

#### 2.1.1 Biomaterials

*Brucella* canis, *Brucella* porcineis, *Brucella* suis, *Brucella* bovis, and related strains were sourced from the China Veterinary Drug Inspection Agency. Staphylococcus aureus, Escherichia coli, Salmonella enteritidis, Shigella, Aspergillus chrysosporium, and Listeria monocytogenes were isolated and cultured by Jiangyin Lingfeng Pet Hospital. Positive and negative blood samples for *Brucella* canis were collected from pet hospitals in Wuxi, Jiangsu Province, China. Additionally, a recombinant plasmid containing the conserved *Brucella* canis Omp25 gene fragment was constructed and preserved in our laboratory.

#### 2.1.2 Main reagents

The Bacterial Genomic DNA Extraction Kit (OMEGA), Plasmid Extraction Kit (Invitrogen), Isothermal Nucleic Acid Amplification Detection Kit (LeShang Biotechnology Co., Ltd.), PCR Product Gel Recovery Kit (Promega), pMD™18-T vectors, dNTPs, Magnesium Chloride, DNA polymerase, ligase, various restriction endonucleases, DL2000 DNA Marker (TaKaRa), B. burgdorferi real-time fluorescence quantitative PCR kit (Beijing Century Yuanheng Animal Epidemiological Defense Technology Co., Ltd.), agarose, TAE electrophoresis buffer (Hyclone), and additional reagents were purchased from Sinopharm Group and Beijing Solepol Science and Technology Co.

#### 2.1.3 Primers and probes

Based on the *Brucella* canis Omp25 gene sequence published in GenBank (accession KC572144.1), conserved gene sequence fragments were selected as templates for primer and probe design. The synthesis of these primers and probes was carried out by Shanghai Sangong Biological Engineering Co.

### 2.2 Methodology

#### 2.2.1 Preparation of plasmid standards

5 mL of blood was aseptically extracted from the medial cephalic vein of the forelimb of sick dogs diagnosed with *Brucella* abortus, and the bacterial genomic DNA was extracted by applying the Bacterial Genomic DNA Extraction Kit, which was used as a template for PCR amplification of the target genes with bp18TOmp25-F and bp18TOmp25-R as primers. Reaction system (50 μL): 5 μL 10×PCR Buffer, 2 μL 50 mM MgCl_2_, 1 μL dNTPs, 2 μL upstream primer, 2 μL downstream primer, 0.5 μL Taq DNA polymerase, 2 μL template, 35.5 μL sterilized ultrapure water; reaction conditions: 95 °C pre-denaturation for 2 min, 94 °C denaturation for 40 s, 48 °C annealing for 40 s, 72 °C extension for 50 s, a total of 2.5 μL of sterile ultrapure water. The reaction conditions: 95°C pre-denaturation for 2 min, 94 °C denaturation for 40 s, 48 °C annealing for 40 s, 72 °C extension for 50 s, a total of 32 cycles, 72 °C continue to extend for 5 min, 4 °C resting for 20 min. PCR reaction products were subjected to 1% agarose gel electrophoresis, the product of the purification of the recovered product after gel cutting, in accordance with the conventional methods of molecular biology for the T-A cloning, and the pMD™ 18-T vector ligation to construct a recombinant plasmid vector, 42 °C thermal excitation method will be the gene carrier into the receptor cells, the ice bath resting 3 min. The gene vector was introduced into the receptor cells by thermal excitation at 42°C, and the cells were allowed to stand for 3 min in an ice bath, then added into the bacterial liquid culture medium and incubated for 2 h at 37°C with shaking at 200 r/min, and then inoculated with culture plates to screen the positive clonal bacterial strains. Then the culture was expanded, plasmid DNA was extracted, and after identification by Ban I digestion and gene sequencing, the correct positive clone strain was identified and conserved, and the concentration of the plasmid DNA genome was determined, the copy number was calculated, and it was stored at -40 °C to be used as a positive plasmid standard, named as bp18TOmp25.

#### 2.2.2 Primer screening

The upstream and downstream primers listed in **Table 1** were paired and combined separately. For the RAA basic assay, 107 copies/μL of positive plasmid standards were utilized as templates. The lyophilized powder of the basic reaction unit was reconstituted by adding 25 μL of reaction buffer, 2 μL of upstream primer, 2 μL of downstream primer, 2 μL of plasmid template, 1 μL of 500 mM magnesium acetate, and 18 μL of sterilized ultrapure water to the reaction tube containing the lyophilized powder. The mixture was then thoroughly mixed and transferred to a 37°C thermostatic water bath for a 30-minute incubation. When the reaction was finished, add 50 μL of 1:1 phenol/chloroform into each tube, then add 50 μL of 1:1 phenol/chloroform to each tube. After the reaction was finished, 50 μL of 1:1 phenol/chloroform mixture was added to each tube, and the DNA was extracted by centrifugation at 12000 rpm for 3 min with sufficient shaking and mixing, and 9 μL of the supernatant was gently aspirated by a micropipette and transferred to another clean centrifugal tube, and 1 μL of 10×Loading Buffer was added, and then the DNA was repeatedly aspirated and mixed, and then it was observed and identified by agarose gel electrophoresis with 1% EB staining solution.

**Table 1:** Primer and probe sequences.

Primer/Probe Name	Sequence (5 ‘-3’)
bp18TOmp25-F	cgcactcttaagtctctcgtaatcg
bp18TOmp25-R	cgcgtcggcatcggctacaagttc
bOmp25-F1	atagctgggctggtggctataccggtctttac
bOmp25-F2	tgggctggtggctataccggtctttaccttg
bOmp25-F3	tggtggctataccggtctttaccttggctac
bOmp25-F4	tataccggtctttaccttggctacggctggaac
bOmp25-F5	ttaccttggctacggctggaacaaggccaagac
bOmp25-R1	gctggtattgccggttcgcagatcaagcttaac
bOmp25-R2	tcacggctggtattgccggttcgcagatcaag
bOmp25-R3	gtacctcacggctggtattgccggttcgcagatc
bOmp25-R4	atgccgtacctcacggctggtattgccggttc
bOmp25-R5	ggttatgccgtacctcacggctggtattgcc
bOmp25-P	atcgtatacggtgttgaaggtgatgcaggttattcctgggccaagaagt

#### 2.2.3 Establishment of RAA fluorescence reaction system

The combination of upstream and downstream primers confirmed by screening was selected as the primers of the RAA reaction system, and the fluorescence reaction system for RAA detection was prepared. Add 25 μL reaction unit buffer, 2 μL upstream primer, 2 μL downstream primer, 1 μL fluorescently labeled probe, 2 μL DNA template, 1 μL 500 mM magnesium acetate, 17 μL sterilized ultrapure water, and a total of 50 μL of the reaction system to the lyophilized powder of the fluorescence reaction unit. After adding all the solutions, the mixture was quickly dissolved and mixed thoroughly, followed by centrifugation at 1500 rpm for 20 seconds. The sample was then transferred to the nucleic acid amplification fluorescence detection instrument, where the temperature was set to 39°C and run for 30 minutes. The fluorescence amplification curve was observed in real-time, and the fluorescence signal values were recorded to determine the results. The negative control was processed using the same method, with sterilized ultrapure water used as the template instead of DNA.

#### 2.2.4 Sensitivity testing of RAAs

The constructed positive plasmid standards were sequentially diluted in 10-fold ratio to form 7 gradient concentrations of sample templates, such as 10^5^ copy/μL, 10^4^ copy/μL, 10^3^ copy/μL, 10^2^ copy/μL, 10 copy/μL, 1 copy/μL, 0.1 copy/μL, etc., and at the same time, we selected the sterilized ultrapure water as the negative control template, and performed sensitivity detection according to the RAA fluorescence reaction system established in 1.2.3. system established in 1.2.3 for sensitivity detection, observe the real-time fluorescence amplification curve, and record and save the fluorescence signal value.

#### 2.2.5 Specific assays for RAA

*Brucella* canis, other different species of *Brucella* porcine, *Brucella* suis, *Brucella* bovis, as well as common canine pathogens such as Staphylococcus aureus, Escherichia coli, Salmonella enteritidis, Shigella, Aspergillus chrysosporium, Listeria monocytogenes, etc., were selected as samples, and the genomic DNA of these bacteria was extracted as the templates of the RAA assay, and then specifically detected in accordance with the established fluorescence reaction system of RAA. The genomic DNA of these bacteria was extracted as the RAA template, and the specific detection was carried out according to the established RAA fluorescence reaction system. Simultaneously, sterilized ultrapure water was used as the negative control template, while a plasmid standard with a concentration of 10³ copies/μL served as the positive control sample. This setup allowed for the assessment of the method’s specificity in detecting *Brucella* canis.

#### 2.2.6 Repeatability tests for RAA

Three different batches of *Brucella* canis positive plasmid standards (10^5^ copy/μL, 10^3^ copy/μL, 10 copy/μL) of the same batch were selected as RAA assay templates, and each sample was assayed 4 times, the fluorescence amplification signals were observed and recorded, and the coefficients of variation within the group were calculated. Meanwhile, four different batches of each concentration of samples were selected as RAA assay templates, and amplification assays were performed separately, and the fluorescence amplification signals were observed and recorded to calculate the intergroup coefficient of variation. The reproducibility of the RAA method for detecting *brucellosis* in affected dogs was assessed through statistical analysis.

#### 2.2.7 Testing of clinical samples

A total of 42 samples were selected as 23 canine *Brucella*-positive blood samples and 19 negative blood samples, which were diagnosed as *Brucella* canis-positive and negative by the testing methods specified in the National Diagnostic Technical Standard for *Brucella* Detection by Bruce Ladder PCR (GB/T 18646-2018), and were inactivated and tested by using commercially available *Brucella* real-time fluorescence PCR kits (Beijing Century-Yuancheng Ltd.) and the RAA method established in this study were used to detect the above 42 blood samples, to compare the significance of the difference between the detection rates of the two, and to test the effectiveness of the RAA method established for the detection of canine *brucellosis* in the clinic.

## 3 Results

### 3.1 Identification of positive plasmid standards

**Figure 1:**
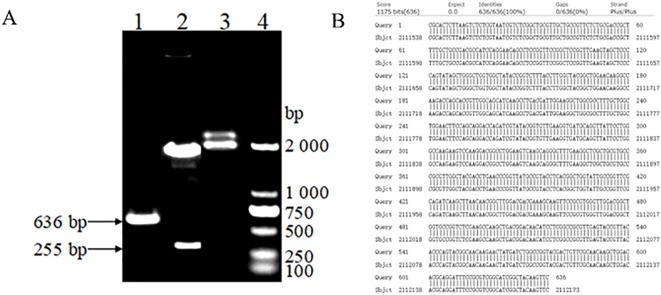
**(A)** Electrophoretic map for the construction and identification of plasmid standards: (1) PCR amplification of the brucella canis Omp25 gene sequence. (2) The recombinant bp18TOmp25 plasmid digested by Ban I restriction endonuclease. (3) The recombinant bp18TOmp25 plasmid. (4) DL2000 DNA Marker. **(B)** Sequencing comparison diagram of plasmid standards.

As shown in **Figure 1**, the electrophoresis band size is 636bp. The constructed bp18TOmp25 plasmid was digested by Ban I restriction endonuclease, and showed a specific electrophoretic band of 255 bp in size on 1% agarose gel electrophoresis, whereas the original p18TC1 plasmid, which had not been digested, did not show this band in electrophoresis, which was in line with the expected results (**Figure 1**). The bp18TOmp25 plasmid was recovered by excision and purification of gel, and the target fragment was sequenced by NCBI. After sequencing, the target fragment was analyzed using BLASTn in the NCBI database. The results indicated that the target gene sequence in the constructed recombinant bp18T-Omp25 plasmid was fully consistent with the conserved region of the Omp25 gene of *Brucella* canis, exhibiting 100% homology. Consequently, the constructed positive plasmid standard was confirmed to be entirely correct.

### 3.2 Primer pairwise screening analysis

**Figure 2:**
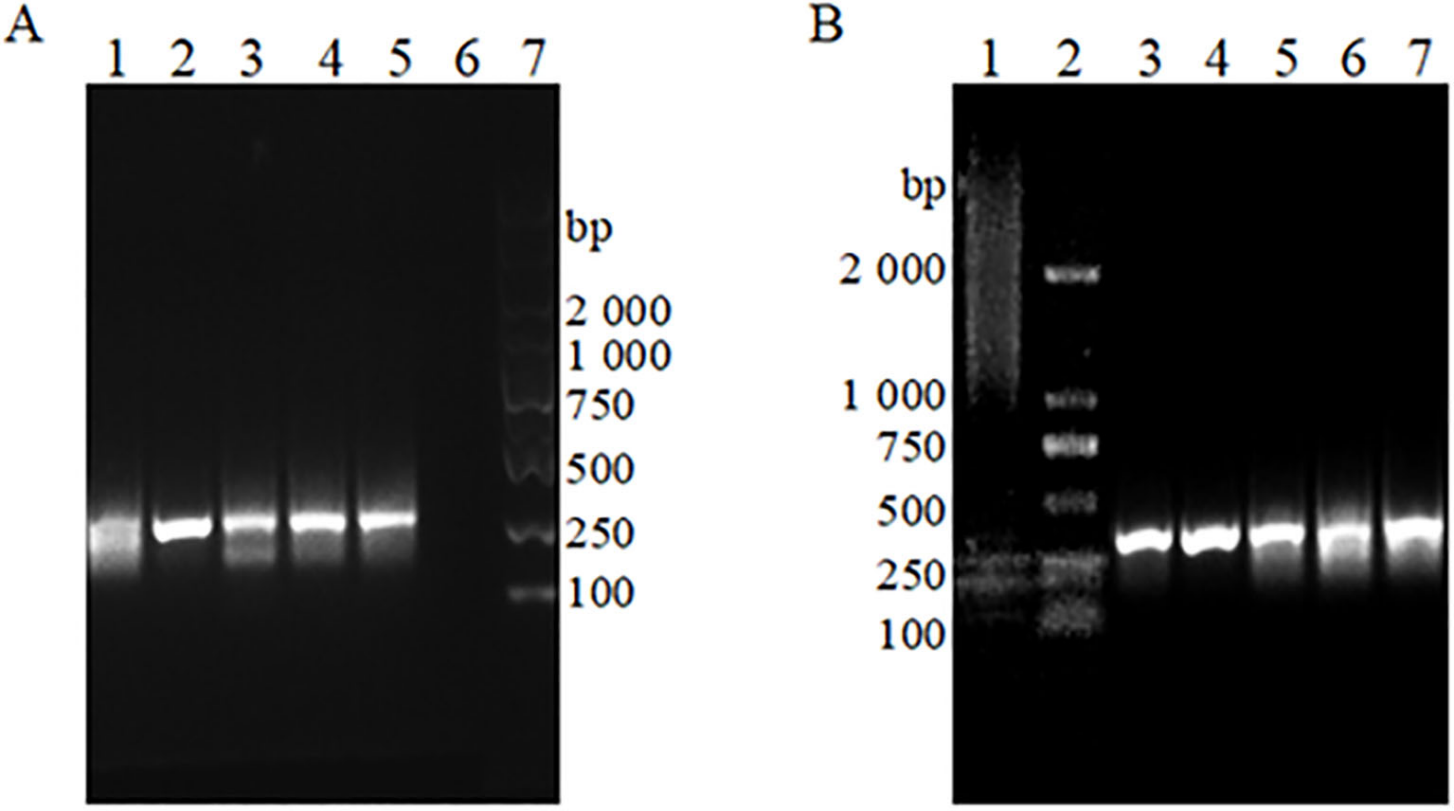
The RAA basic detection gel electrophoresis patterns of different primer pairs **(A)** Paired combinations of upstream primer bOmp25-F1 with different downstream primers. 1: Primer bOmp25-F1/bOmp25-R1 combination; 2: Primer bOmp25-F1/bOmp25-R2 combination; 3: Primer bOmp25-F1/bOmp25-R3 combination; 4: Primer bOmp25- F1/bOmp25-R4 combination; 5: Primer bOmp25-F1/bOmp25-R5 combination; 6: No primers; 7: DL2000 DNA Marker. **(B)** Downstream primer bOmp25-R2 is paired with different upstream primers. 1: No primers; 2: DL2000 DNA Marker; 3: Primer bOmp25-F1/bOmp25-R2 combination; 4: Primer bOmp25-F2/bOmp25-R2 combination; 5: Primer bOmp25-F3/bOmp25-R2 combination; 6: Primer bOmp25-F4/bOmp25-R2 combination; 7: Primer bOmp25-F 5/bOmp25-R2 combination.

Positive plasmid standards at a concentration of 10^7^; copies/μL were utilized as sample templates. The results of the RAA-based assays, screened with various combinations of upstream and downstream primers, are presented in **Figure 2**. First, the upstream primer bOmp25-F1 was used to form a matrix primer combination with the downstream primers bOmp25-R1, bOmp25-R2, bOmp25-R3, bOmp25-R4, and bOmp25-R5, respectively, to perform the primer screening for the RAA assay, and the analysis of 1% agarose gel electrophoresis profiles showed that (**Figure 2A**), except for the samples with no added primer that did not amplify Except for the sample without added primer which did not amplify any bands, the rest of the various primer pair combinations could amplify obvious bands, among which the primer bOmp25-F1/bOmp25-R2 pair combination was more effective in RAA detection, with the brightest electrophoretic bands, high sensitivity, no smearing phenomenon, and high specificity. Then the downstream primer bOmp25-R2 with better amplification effect was selected to pair-combine with the upstream primers bOmp25-F1, bOmp25-F2, bOmp25-F3, bOmp25-F4, and bOmp25-F5, respectively, and primer screening for RAA detection was carried out again, and the analysis of 1% agarose gel electrophoresis profiles showed that (**Figure 2B**), the samples with no primers did not The remaining primer combinations were able to amplify obvious specific bands, among which the primer bOmp25-F2/bOmp25-R2 pair combination amplified better, with brighter bands, no smearing phenomenon, and the best sensitivity and specificity. The results indicated that the primer combination bOmp25-F2/bOmp25-R2 identified in this experiment was the most effective for RAA detection of *Brucella* canis and can be utilized for establishing the subsequent RAA fluorescence reaction system.

### 3.3 Sensitivity analysis of RAA detection methods

**Figure 3:**
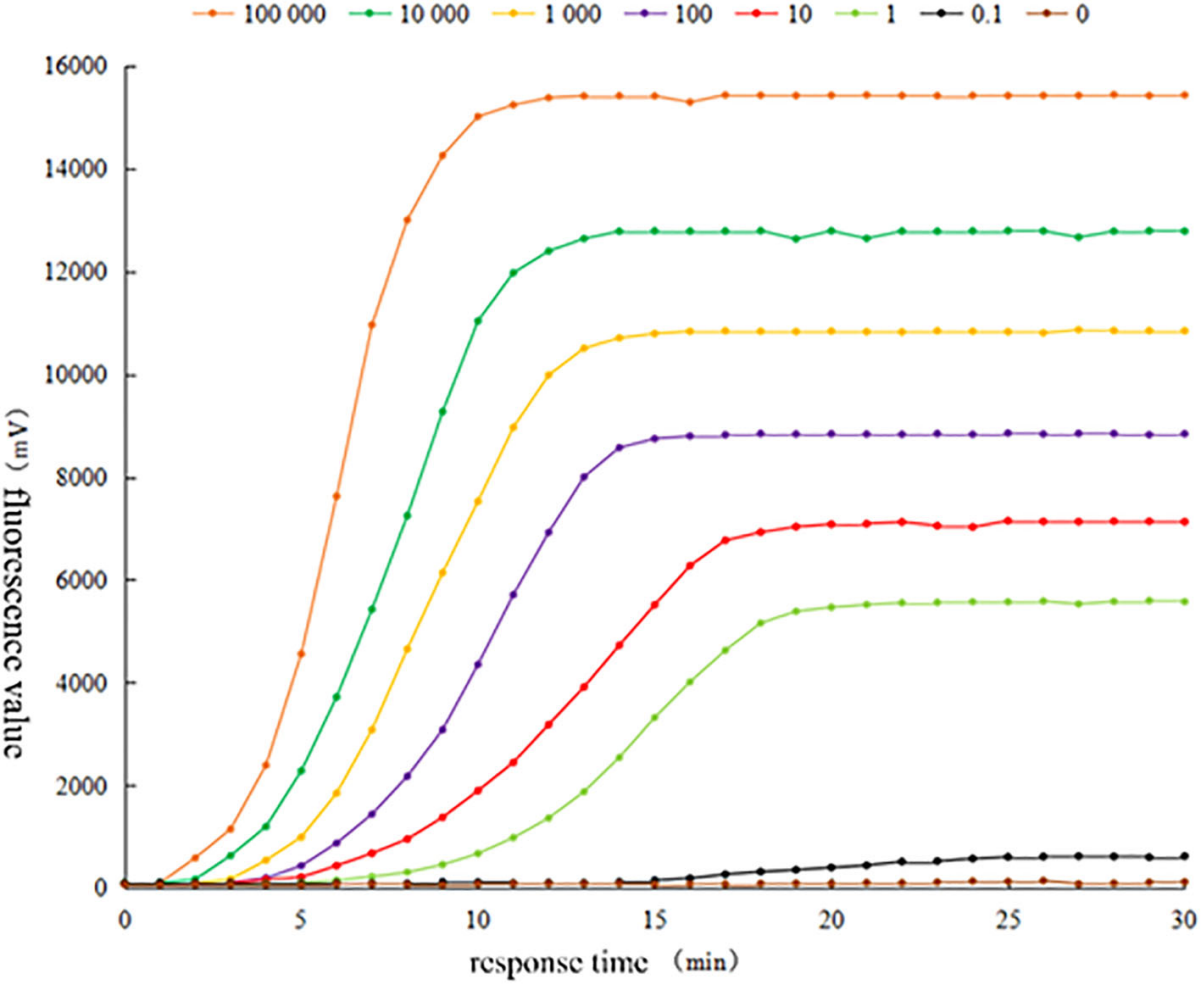
Sensitivity test of RAA fluorescence method for detecting Brucella canis.

The fluorescence curves of RAA assay by using the positive plasmid standard containing *Brucella* canis Omp25 gene sequence as a sample template after 10-fold gradient dilution are shown in **Figure 3**. The fluorescence curves of RAA assay were observed within 15 min at the concentrations of 10^5^ copies/μL, 10^4^ copies/μL, 10^3^ copies/μL, 10^2^ copies/μL, 10 copies/μL, 1 copy/μL, respectively. However, the peak intensity of the fluorescence curve gradually decreased with less sample concentration, and the peak onset time was delayed accordingly. However, the peak intensity of the curve decreased with the lower sample concentration, and the peak onset time was delayed accordingly. No obvious fluorescence curves were observed in the samples with a concentration of 0.1 copies/μL and sterilized ultrapure water by RAA. The results showed that the lowest detection limit of the method based on RAA technology for the rapid detection of *Brucella* canis was 1 copy/μL, i.e., the detection sensitivity was 1 copy/μL.

### 3.4 Specificity analysis of RAA detection methods

**Figure 4:**
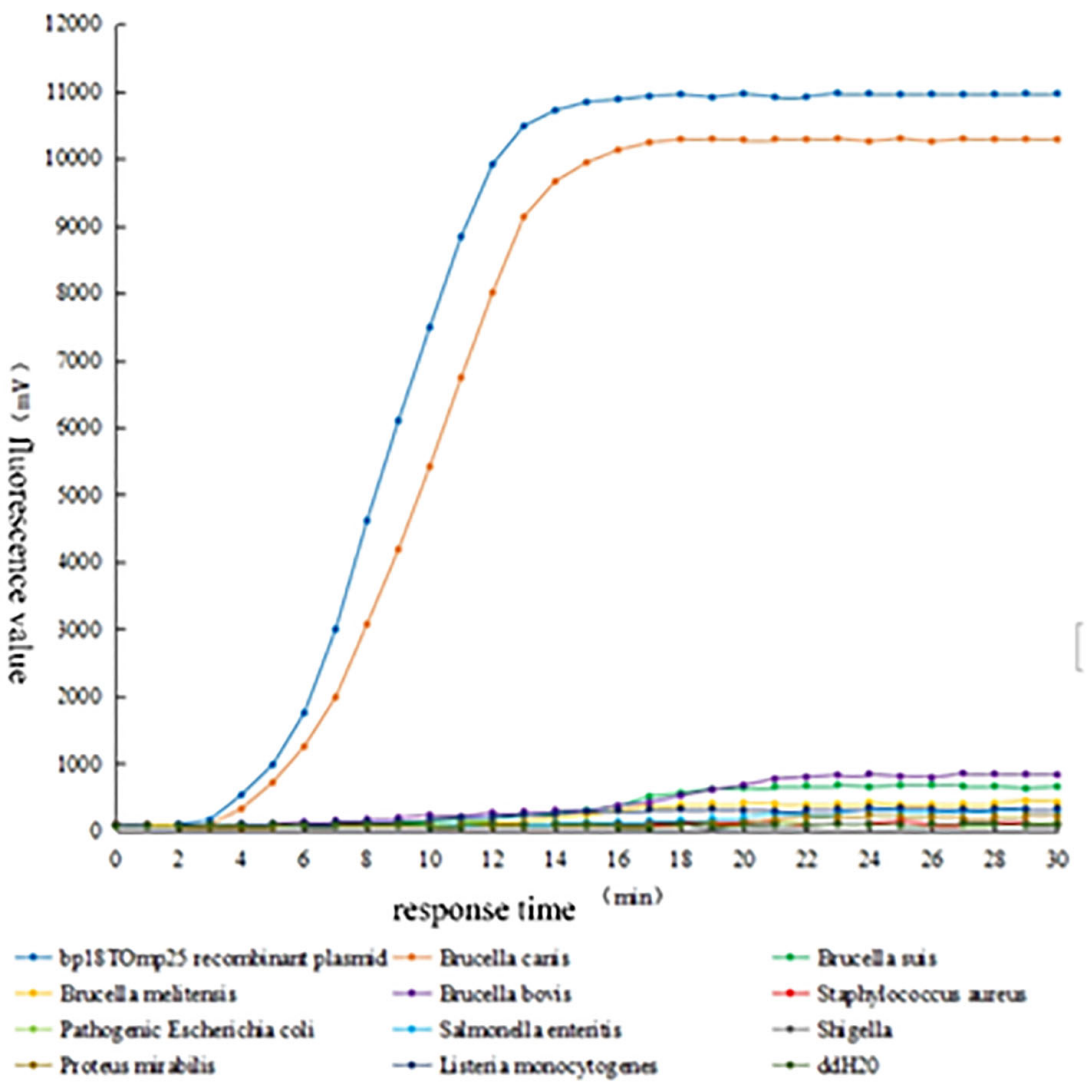
Specificity test of RAA fluorescence method for detecting Brucella canis.

The genomic DNA of *Brucella* canis, *Brucella* porcine, *Brucella* suis, *Brucella* bovis, Staphylococcus aureus, Escherichia coli, Salmonella enteritidis, Shigella, Aspergillus chrysosporium, Listeria monocytogenes and 10^3^ copies/μL of the positive plasmid standard and sterilized ultrapure water were selected as sample templates, and detected in accordance with the established RAA fluorescence system, as can be seen in the fluorescence curves in **Figure 4**. From the fluorescence curve shown in **Figure 4**, it is evident that both *Brucella* canis and the positive plasmid standard exhibited distinct fluorescence curves within 15 minutes, while the other samples and sterilized ultrapure water did not demonstrate significant fluorescence. These results indicate that the RAA method established in this experiment possesses good specificity for detecting *Brucella* canis, effectively amplifying its genomic DNA without cross-reactivity with other *Brucella* species or common clinical pathogens in canines.

### 3.5 Repeatability analysis of RAA detection methods

The intra- and inter-batch reproducibility of the RAA method was assessed using positive plasmid standards at three concentration gradients: 10^5^ copies/μL, 10³ copies/μL, and 10 copies/μL. As shown in **Table 2**, the intra-group coefficients of variation (CV) for the detection of *Brucella* canis using the RAA method were all below 1.00% (CV values of 0.97%, 0.95%, and 0.49%, respectively), indicating excellent repeatability within the same batch. Additionally, the inter-group coefficients of variation for *Brucella* canis detection by RAA were less than 2.00% (CVs of 1.53%, 1.21%, and 0.91%), demonstrating good reproducibility of the test across different batches.

**Table 2:** Intragroup and intergroup repeatability tests for RAA fluorescence detection of *Brucella*.

(copies/μL)	n	Intra-assay		Inter-assay	
		`x ± s	CV(%)	`x ± s	CV(%)
10^5^	4	15479 ± 149	0.97	15370 ± 234	1.53
10^3^	4	10796 ± 103	0.95	10867 ± 131	1.21
10	4	7155 ± 35	0.49	7160 ± 65	0.91

### 3.6 Clinical sample validation

In this study, both the RAA method and a commercially available real-time fluorescence quantitative PCR method were employed to test 23 positive and 19 negative canine serum samples. The results revealed that the real-time fluorescence quantitative PCR method detected 20 positive samples, yielding a detection rate of 86.86%. In contrast, the RAA method identified all 23 positive samples, achieving a detection rate of 100%. The compliance rate for positive detection with the RAA method was significantly higher than that of the commercially available real-time fluorescence quantitative PCR method (P < 0.05), as illustrated in **Table 3**.

**Table 3:** Comparison of clinical sample validation results [n (%)].

Group	n	positive	negative
		23	19
Fluorogenic quantitative PCR	42	20(86.96)	22(115.79.00)
RAA	42	23(100.00)	19(100.00)
χ²		0.429	
P		0.001	

## 4 Discussion

Dogs are among the first mammals to be domesticated by humans and currently represent the most widely bred pets globally. According to United Nations statistics, the global dog population is approximately 600 million, with around 200 million dogs bred in China alone (Aggarwal et al., 2022; Mao and Liu, 2023). Because it has always been loved by people, the diagnosis and prevention of related canine epidemics have gradually received extensive attention from all walks of life. Canine *brucellosis* is a type B animal infectious disease caused by *Brucella* infection. Once the dog is infected, it often leads to abortion, stillbirth, infertility and other reproductive disorders and fever, joint damage and other clinical features (Ishihara et al., 2024).In recent years, with the increasing number of dogs, the incidence of this disease and the mortality rate have been on the rise year by year, and the degree of public harm is more serious (Tymczak et al., 2022).In addition, canine *brucellosis* is mostly hidden, not easy to be detected, once there are obvious clinical symptoms, the disease has developed to a more serious stage (Camargo-Castañeda et al., 2021; Hamdy et al., 2023). Therefore, actively carrying out early clinical correct diagnosis for the prevention and treatment of the disease plays a crucial role. At present, in addition to the identification of clinical symptoms and pathogenic bacteria isolation and culture, ELISA, immunofluorescence detection, PCR and other methods commonly used in the laboratory for the detection of *brucellosis* in dogs, there are low sensitivity, poor specificity and low reliability of differential diagnosis, making it very easy to misdiagnosis, omission of the diagnosis, but also time-consuming and laborious, and it is difficult to promote the application of the field in the rapid detection (Laverde et al., 2021; Lopes et al., 2022; Yan et al., 2022).Therefore, the establishment of a rapid, highly sensitive and specific accurate clinical detection method is of great clinical value for the correct diagnosis and precise prevention and control of this disease. The RAA method successfully established in this study has achieved preliminary good test results in the rapid detection of canine *brucellosis*, which provides implications for the subsequent diagnosis of canine diseases.

With the ongoing advancement of modern diagnostic technologies and life sciences, conventional PCR methods are increasingly inadequate for meeting the current demands of animal disease detection. In recent years, recombinant enzyme-mediated constant-temperature nucleic acid amplification detection technology has emerged as a promising alternative to traditional PCR and fluorescent quantitative PCR. This innovative technology offers several advantages, including constant temperature operation, ease of use, reduced detection time, high sensitivity, and strong specificity (Sun et al., 2024; Xiao et al., 2021; Luan et al., 2024), which has brought a new dawn to the industry of animal disease detection, and is widely favored by many domestic and foreign detection workers and clinical veterinarians. At present, there have been some relevant literature reports in this area, for example (Paenkaew et al., 2023), established a recombinant enzyme polymerase-mediated thermostatic amplification method for the detection of canine rickettsial pathogens, based on the method of the detection of a constant temperature of 37 °C, the detection time of 20 min, the sensitivity reaching 100 copies, and there is no cross-reactivity with other pathogens (Rong et al., 2023). established a recombinant enzyme polymerase-mediated thermostatic amplification method for the detection of canine rickettsiae pathogens. Rong Y et al. developed a recombinant enzyme polymerase-mediated rapid amplification method for the detection of Trichomonas canis at a constant temperature, with a minimum detection limit of 100 copies/µL, which is 100 times more sensitive than the traditional PCR method and has no cross-reactivity with the genomes of Giardia duodenalis and other parasites, and it is worthwhile to popularize the use of this method because of its simplicity, sensitivity, and specificity when compared with the common polymerase chain reaction (Coertse et al., 2019). The study on the detection of the rabies virus using reverse transcription recombinase polymerase amplification demonstrated a sensitivity of 562 RNA copies, with a rapid detection time of only 20 minutes and high specificity. Furthermore, the conformity rate of clinical samples verified for rabies virus diagnosis reached 100%. However, the application of RAA detection technology in the diagnosis of canine *brucellosis* has not yet been reported in the literature, either domestically or internationally.

Currently, in the field of bacterial molecular diagnosis and nucleic acid amplification detection, 16S rRNA, as a kind of ribosomal RNA of prokaryotes, is often used as a molecular indicator, and has now become a powerful tool for microbial detection and differential diagnosis due to the conservativeness of its gene sequences and the ubiquity of its existence (Bertolo et al., 2024; Bonanno Ferraro et al., 2024). However, there are some problems such as differences in gene amplification efficiency, multicopy heterogeneity, horizontal gene transfer, etc., which are not favorable for molecular amplification detection. The Omp25 gene, a member of the OmpA family, is an important outer membrane protein of *Brucella*, which plays an important role in maintaining the structural stability of the bacterial outer membrane (Lopes et al., 2022). Some studies have shown that the nucleotide sequence of the Omp25 gene is highly conserved, and the sequence variability is large among different strains and genera (Elrashedy et al., 2024). In this study, we selected the outer membrane protein Omp25 gene sequence of *Brucella* canis as the target gene for constructing recombinant plasmid standards and for designing primers and probes. We ultimately established a recombinant enzyme-mediated thermostatic nucleic acid amplification method for detecting *Brucella* canis, utilizing the primers bOmp25-F2 and bOmp25-R2, along with the probe bOmp25-P. The reaction was conducted at a constant temperature of 39°C for 15 minutes, achieving a detection sensitivity of 1 copy/μL. Importantly, there was no significant cross-reactivity observed with various *Brucella* species, including *Brucella* canis, *Brucella* suis, and *Brucella* bovis, as well as common clinical pathogens such as *Staphylococcus aureus, Escherichia coli, Salmonella enteritidis, Shigella, Aspergillus chrysosporium, and Listeria monocytogenes*. Additionally, the coefficient of variation for the intra-batch reproducibility test was less than 1.00%, while the inter-batch reproducibility test yielded a coefficient of variation of less than 2.00%. Regarding the molecular detection methods of *Brucella* canis, at present, it still mainly relies on the principle of PCR technology, for example, Ye YB et al (Ye et al., 2022) applied the PCR method for the detection of *Brucella* canis, and the results showed that its detection sensitivity was 100 copies, and the detection time was 2 h, which had a certain reasonable specificity. Mol JPS et al (Mol et al., 2020) compared the detection of *Brucella* canis between different serological detection methods and the PCR method, and the results showed that the PCR detection was more sensitive than the PCR method. Mol JPS et al. compared the detection of *Brucella* canis by different serologic tests with PCR, and the results showed that the positive rate of PCR was higher than that of serologic tests, but there still existed the phenomenon of leakage caused by the lack of sensitivity. The sensitivity and other properties of these PCR methods for the detection of *Brucella* canis were much lower than the results of the RAA test established in this study. This fully demonstrates that the RAA method established in this study for the detection of *Brucella* canis has good performance, high sensitivity, high specificity and good reproducibility, which is significantly better than other related similar literature reports at home and abroad (Camargo-Castañeda et al., 2021; Süer et al., 2023; Mol et al., 2020; Ye et al., 2022; Qin et al., 2019; Hamdy et al., 2023; Nan et al., 2018; Kang et al., 2014; Boeri et al., 2018).

In this study, we employed the matrix screening method for primer selection. This approach involved initially selecting one upstream primer (bOmp25-F1) and screening it against various downstream primers in pairwise combinations. Subsequently, we conducted pairwise combinations with different upstream primers, ultimately identifying the optimal primer combination (bOmp25-F2/bOmp25-R2) after only 10 pairwise combinations. This streamlined the operational process, resulting in reduced labor and cost. Additionally, we opted to construct a recombinant vector plasmid as a positive standard due to its advantageous properties, including a small relative molecular weight, ease of isolation and manipulation, independent replication without reliance on the nuclear genome, the ability to carry numerous copies, and stable performance (Xi et al., 2022, Kieu Doan and Croyle, 2023, Liu et al., 2024).Based on this, we chose to construct a recombinant vector plasmid as a standard and diluted it with different gradient concentrations as a template for the detection of RAA, and successfully established the RAA assay for *Brucella* canis. In this study, we successfully established the reaction system and technical method for the detection of *Brucella* canis by RAA. The positive detection compliance rate of the RAA method was significantly higher than that of the commercially available real-time fluorescence quantitative PCR method.

In conclusion, this study successfully established a method based on recombinant enzyme-mediated thermostable nucleic acid amplification technology for the rapid detection of *Brucella* canis. This method offers several advantages, including rapid detection, high sensitivity, strong specificity, and excellent reproducibility, making it a valuable clinical rapid detection kit. It provides a novel approach for the swift detection of canine *brucellosis* and aids clinical veterinarians in the accurate and rapid diagnosis of other pet-related epidemics. The method demonstrates significant potential for widespread application and dissemination in veterinary practice.

## 5 Limitations

Although our research is innovative, it has the following limitations. First, in this study, we used the RPA method to detect Brucella Canis, and the samples collected were mainly from Wuxi City at present. This work has not been carried out for the time being for the detection and application of samples from other areas of the country or the world that may have quality, environment, pathogen strain variation and other conditions. In the later stage, we will expand the sample collection area, collect samples of different tissue types and increase the number of samples to study more diverse sample groups. Second, population sample detection is also a direction worthy of further research in the future. Third, The requirements of RPA detection on environmental conditions and operating conditions are not too high initially, and it is very promising to become a rapid on-site detection method.

## Data Availability

The raw data supporting the conclusions of this article will be made available by the authors, without undue reservation.
